# Eco-Friendly Alternatives to Toluene-Based 2D Inks for Inkjet and Electrohydrodynamic Jet Printing Processes: A Rheological Study

**DOI:** 10.3390/mi16020130

**Published:** 2025-01-23

**Authors:** Pedro C. Rijo, Ilaria Tocci, Francisco J. Galindo-Rosales

**Affiliations:** 1 CEFT—Transport Phenomena Research Center, Mechanical Engineering Department, Faculty of Engineering, University of Porto, Rua Dr. Roberto Frias, 4200-465 Porto, Portugal; 2 ALiCE—Associate Laboratory in Chemical Engineering, Faculty of Engineering, University of Porto, Rua Dr. Roberto Frias, 4200-465 Porto, Portugal; 3 DICMaPI—Dipartimento di Ingegneria Chimica, dei Materiali e della Produzione Industrial, Università degli Studi di Napoli Federico II, P.le V. Tecchio 80, 80125 Napoli, Italy; 4 Department of Engineering, University of Campania “Luigi Vanvitelli”, Via Rome 29, 81031 Aversa, Italy; 5 CEFT—Transport Phenomena Research Center, Chemical Engineering Department, Faculty of Engineering, University of Porto, Rua Dr. Roberto Frias, 4200-465 Porto, Portugal

**Keywords:** 2D particles, rheology, printing processes

## Abstract

Green sustainable solvents have emerged as promising alternatives to petroleum-derived options, such as toluene. This study demonstrates the use of cyrene as an effective exfoliation medium for graphene nanoplatelets (GNPs) and hexagonal boron nitride (hBN) and molybdenum disulfide (MoS_2_) particles. The incorporation of polyvinylpyrrolidone (PVP) attenuates the shear-thinning behavior of GNP and hBN suspensions, maintaining a constant shear viscosity over a wide range of shear rates regardless of PVP molecular weight. Despite the presence of polymer, elasticity is hindered by inertia effects, making it impossible to accurately measure the extensional relaxation time in the capillary breakup extensional rheometer (CaBER). Assuming the weak elasticity of the formulations has a negligible impact on the breakup mechanism, we estimated droplet sizes for drop-on-demand (DoD) inkjet printing and electrohydrodynamic (EHD) jet printing based on fluid properties, i.e., viscosity, surface tension and density, and nozzle inner diameter ($D_{nozzle}$). Results indicate that the droplet size ratio ($D_{drop}/D_{nozzle}$) in DoD printing can be up to two orders of magnitude higher than the one predicted for EHD jet printing at the same flow rate. This work highlights the potential of cyrene-based 2D inks as eco-friendly alternatives for advanced printing technologies.

## 1. Introduction

Two-dimensional nanomaterials as functional materials have received a lot of interest due to their applicability in areas such as flexible electronics, energy storage, biomedical applications, etc. [1,2,3,4,5,6,7]. The most widely used nanomaterials have been graphene, molybdenum disulfide (MoS_2_) and hexagonal boron nitride (hBN) due to their distinct mechanical, optical and electrical properties [8]. These nanomaterials allow the formulation of conductive inks or dielectric inks. However, their properties are only obtained after exfoliation.

Liquid-phase exfoliation (LPE) has been the most widely used method for exfoliating 2D particles [9,10]. This technique makes it possible to produce a great number of nanosheets with good control of the thickness and size of the flakes and with a low degree of defects compared to other mechanical means. Furthermore, LPE has proven to be a simple and inexpensive alternative to hydrothermal and chemical vapor deposition techniques in the production of defect-free nanosheets [9].

The success of LPE in obtaining nanosheets and their dispersion in the solvent depends on several factors: polarity, viscosity, surface tension, and, recently, toxicity and sustainability in the production of the exfoliating liquid [1,10]. According to Gravelle et al. [10], particle exfoliation is better when the surface tension of the liquid is closer to the surface tension/surface energy of the particles to be exfoliated. It also depends on the viscosity of the solvent, i.e., the higher the viscosity of the solvent, the better the dispersion of the particles and the lower their mobility to aggregate again. Salavagione et al. [1], Franco et al. [11], Tkachev et al. [12], Adam et al. [9], Occhiuzzi et al. [13] and Martínez-Jiménez et al. [3] observed that n-methyl-2-pyrrolidone (NMP) and dimethylformamide (DMF) are the solvents that produce a great number of nanosheets of graphene, hBN and MoS_2_ through liquid-phase exfoliation. This happens since the surface tension of the solvents is very close to the surface energy of the particles (Table 1).

As NMP and DMF are very toxic solvents for both human health and the environment, they are included in the European Chemical Agency (ECHA) blacklist [1,15]. Any ink based on NMP or DMF can only be marketed with special authorization from the ECHA. To overcome these restrictions, washing and drying the exfoliated particles in NMP or DMF is an intermediate step, followed by redispersion of the dried particles without any residual traces of NMP or DMF in carrier fluid with the appropriate rheological properties for a given printing technique. Usually, the exfoliated particles are dispersed in water-based or alcohol-based dispersing fluids [16].

Recently, Rijo et al. [8] used toluene and toluene/ethanol as exfoliating liquids in the production of hBN-inks and MoS_2_-inks for electrohydrodynamic (EHD) jet printing. The use of toluene enables the production of nanoparticles composed of a few layers; moreover, the addition of ethyl cellulose has significantly improved the stability of the dispersion and reduced the mobility of the particles to reaggregate because of the viscosity of the polymer solution. On the other hand, toluene is a solvent that promotes the release of volatile organic compounds (VOCs), which are harmful to the environment and toxic to humans when exposed for long periods of time [2]. These disadvantages make the use of toluene as a solvent-based ink slightly unpopular.

Seeking to overcome the environmental issues imposed during ink formulation, Salavagione et al. [1], Franco et al. [11], Tkachev et al. [12] and Pan et al. [17] proposed the use of dihydrolevoglucosenone, commercially known as cyrene, as an exfoliation liquid for graphene particles. Cyrene is a pale yellow, aprotic, dipolar, green solvent derived from cellulose biomass [9]. Cyrene has a viscosity of 14.5 mPa·s and a surface tension ranging from 33.6 mN/m (Sigma-Aldrich, Lisbon, Portugal) to 72.5 mN/m (Circa Group, Oslo, Norway), depending on the manufacturer [9,18]. As the surface tension of cyrene is close to the surface energy of graphene, and the viscosity is an order of magnitude higher than the viscosity of NMP or DMF, it was possible to produce a double concentration of graphene nanosheets. Moreover, the use of cyrene made it possible to produce nanosheets with a lower degree of defects compared to the nanosheets produced in NMP, which improves the electrical conductivity of the nanosheets [1,11,12,17].

Saving time in washing and drying the exfoliated particles, Franco et al. [11] and Pan et al. [17] used cyrene as the base solvent in the production of conductive inks for screen printing. They were successful in printing functional graphene-based electric circuits. However, they found that a binder needed to be added to improve the adhesion of the nanosheets to the substrate. The addition of polymer must be properly studied to meet the ink viscosity and surface tension standards appropriate for printing techniques [19,20,21]. Regarding the MoS_2_ particles, these particles were easily exfoliated in cyrene, promoting the production of higher-quality nanosheets compared to nanosheets produced in NMP or DMF [9,13].

So far, cyrene has been used in the formulation of screen-printing inks. Despite the low production costs and high throughput rates of screen printing, this technique becomes expensive or impractical when the scale of the printing is reduced, i.e., printing micro or nanodevices. As an alternative, inkjet printing and electrohydrodynamic (EHD) jet printing have proven to be viable for printing devices with high resolution and precision at the micro- and nanoscale using 2D nanomaterials [22,23,24,25].

Ink formulation is a complex process that consists of mixing several components: functional material, polymeric resin/binder, additives and solvent [26]. The concentration of each component varies from printing process to printing process. Screen printing requires pasty inks (solvent concentration of 15–65% *w*/*w*) whose viscosity varies between 500 mPa·s and 5000 mPa·s, while inkjet printing and EHD jet printing require fluid inks (solvent concentration of 60–90% *w*/*w*) whose viscosity varies between 1 and 30 mPa·s for inkjet printing and 1 and 4000 mPa·s for EHD jet printing [19,20,26]. In terms of surface tension, the three printing techniques require the same range of values: 15–50 mN/m [19,20,26].

In inkjet printing, the printing is perfect when the inks have an Ohnesorge number between 0.1 and 1, a Weber number between 2 and 25 and a Reynolds number ranging between 2 and 200 [19,24]. If these conditions are respected, the ink has enough energy to form a droplet without the need to form satellite droplets after detaching it from the printer head and avoids the droplet splashing when it hits the substrate [19,24]. In EHD jet printing, the inks must have a minimum electrical conductivity that allows the meniscus, which is attached to the needle tip, to deform into a structure like a Taylor cone [27,28]. This happens because of the movement of the electrical charges inside the fluid to the meniscus’ surface and then to the counter electrode due to the application of an electric field. While in inkjet printing the droplet diameter can be several times larger than the nozzle diameter [29], in EHD jet printing, the droplet or jet diameter can be an order of magnitude smaller than the nozzle diameter [27,28].

Considering the rheological characterization of toluene-based 2D inks reported in previous works [8,30,31,32], the authors propose to formulate greener and more sustainable 2D inks by replacing toluene with cyrene. Thus, the inks are composed of 2D nanoparticles with different electrical conductivities (graphene, MoS_2_ and hBN) that are exfoliated and dispersed in cyrene. Generally speaking, the addition of polymer may improve not only the stability of the suspension but also increase the viscosity of the ink and may improve its adhesion of the particles to the substrate [24]. Polyvinylpyrrolidone (PVP) is a very versatile non-ionic polymer and has been employed in many different fields, but it is particularly well known in the pharmaceutical industry for its superior properties regarding inertness, non-toxicity and biocompatibility [33]. Moreover, the biodegradation of PVP can be enhanced when combined with a co-substrate and the fungus Penicillium chrysogenum [34].

In this article, authors will study how the addition of PVP to the cyrene-based formulation may affect the viscoelasticity and electrical conductivity of the suspension, which are key parameters for its printability by inkjet printing techniques and EHD jet printing processes.

## 2. Materials and Methods

### 2.1. Materials

Cyrene, polyvinylpyrrolidone (PVP) with different molecular weights (*MW* = 10,000 g/mol and *MW* = 40,000 g/mol), molybdenum disulfide (particle size < 2 μm) and boron nitride (particle size < 2 μm) were purchased from Sigma-Aldrich (Lisbon, Portugal). Graphene nanoplatelets with a particle size distribution of 1 to 20 μm were supplied by Graphenest (Sever do Vouga, Portugal). All chemicals were used as received. The PVP polymer solutions were prepared by dissolving PVP in cyrene using a roller mixer for 48 h. The concentrations of PVP ranged from 0.5% *w*/*w* to 5.0% *w*/*w* for *MW* = 10,000 g/mol and from 0.5% *w*/*w* to 2.5% *w*/*w* for *MW* = 40,000 g/mol. The graphene, MoS_2_ and hBN particles were dispersed and exfoliated in cyrene and in two PVP solutions of different molecular weights for 3 h using an ultrasonic bath. A detailed description of the protocol used to prepare the suspensions can be found at [30,31].

### 2.2. Particle Size and Zeta Potential

The particle size of GNP, MoS_2_ and hBN after dispersion and exfoliation in the dispersant medium was evaluated by a dynamic light scattering (DLS) technique using Malvern Zetasizer Nano ZS equipment (Malvern Panalytical, Porto, Portugal). For these measurements, a dilution factor of 100 for the particle concentration was used to obtain semi-transparent suspensions, avoiding errors during the measurements. The dispersant mediums used here were cyrene and two polymer solutions of PVP dissolved in cyrene with different molecular weights.

The zeta potential of GNP, MoS_2_ and hBN in cyrene was assessed by laser Doppler electrophoresis using the same equipment for DLS. Moreover, how the dissolved polymer affects the zeta potential of particles was also studied.

Additionally, the exfoliation of the nanoparticles in different solvents was also analyzed using a Transmission Electron Microscope (Jeol JEM 1400, Joel SAS, Croissy-sur-Seine, France) to evaluate their dispersion and structural characteristics.

### 2.3. Measurements of Electrical and Physical Properties

A Biolin Sigma 700 tensiometer (Biolin Scientific UK, Manchester, UK) was used to measure the physical properties of fluids using the platinum Du Nouy ring for measuring surface tension and the density probe for the density measurements.

The electrical conductivity was determined by applying a voltage difference between the ends of a borosilicate capillary filled with fluid. Electrical conductivity was obtained from the slope of the linear relationship between the applied voltage and measured electric current. The protocol used is the same as the one used in [35,36].

All experiments were done at an ambient temperature of 20 °C.

### 2.4. Rheological Characterization

A stress-controlled rotational rheometer (Anton Paar MCR301, Anton Paar GmbH, Graz, Austria) was used to measure the steady shear viscosity (η) of fluids for a shear rate ($\dot{\gamma }$) range of 1 to 10^5^ s^−1^. A plate-plate geometry of 50 mm in diameter and a gap of 0.250 mm were used to perform the shear rheological characterization of fluids; moreover, all experiments were done at a temperature of 20 °C controlled by a Peltier cell. For each fluid, five independent runs were needed to ensure the reliability of the results.

A capillary breakup extensional rheometer (Thermo HAAKE CaBER-1, Thermo Fisher Scientific, Waltham, MA, USA) coupled with a high-speed camera (Photron FASTCAM Mini UX 100, West Wycombe, Buckinghamshire, UK) was used to study the filament thinning process of each fluid, as well as its breakup process. Digital images of the filament thinning and breakage processes were recorded at 20,000 frames per second. A detailed description of the experimental setup used here can be found in [30]. Additionally, all experiments were conducted at 20 °C, ensuring the reliability of the results.

## 3. Results and Discussion

### 3.1. Characterization of the Nanomaterials

**Figure 1:**
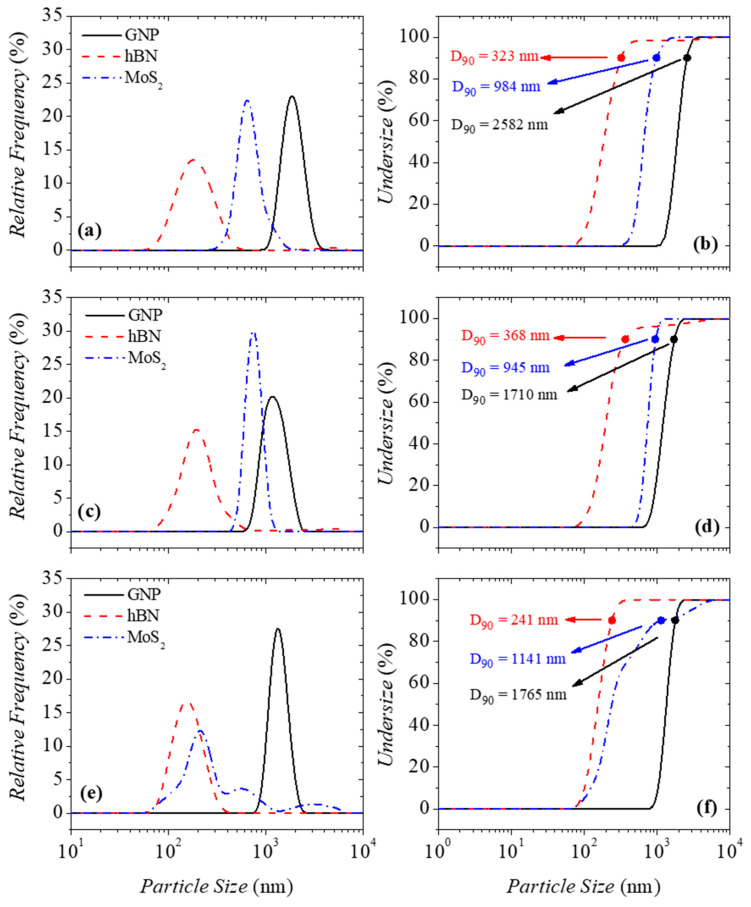
Relative frequency and undersize DLS curves of nanomaterials dispersed (**a**,**b**) in cyrene, (**c**,**d**) in PVP10 dissolved in cyrene with a concentration of 2.5% *w*/*w* (Cyr-PVP10-2.5), and (**e**,**f**) in PVP40 dissolved in cyrene at a concentration of 0.75% *w*/*w* (Cyr-PVP40-0.75).

The size of the particles after the liquid-phase exfoliation (LPE) was measured by dynamic light scattering (DLS). Figure 1 shows the relative frequency and cumulative undersize DLS curves of the nanomaterials studied here. In this work, there was the opportunity to evaluate how the presence of dissolved polymer affects the exfoliation process of the particles. According to the particle suppliers’ technical data sheet, the hBN and MoS_2_ particles have a size below 2 μm, while the GNP particles have a size distribution range between 1 and 20 μm before the exfoliation process.

Independently of the presence of polymer, the cyrene reveals itself to be a good exfoliation solvent for hBN particles, followed by MoS_2_ particles and GNP particles. Table 2 shows the average hydrodynamic diameter of the particles after the exfoliation process, and it is possible to observe that the particles are partially exfoliated.

Furthermore, 90% of the exfoliated hBN particles have a size ($D_{90}$) up to 368 nm, and the $D_{90}$ parameter increases to 1141 nm for MoS_2_ and 1765 nm for GNP (Figure 1). When GNP exfoliation occurs without the presence of polymer, the $D_{90}$ increases to 2582 nm. Despite the size detected for GNP particles by DLS, this agrees with the size of graphene measured by Salavagione et al. [1] using transmission electron microscopy (TEM).

**Figure 2:**
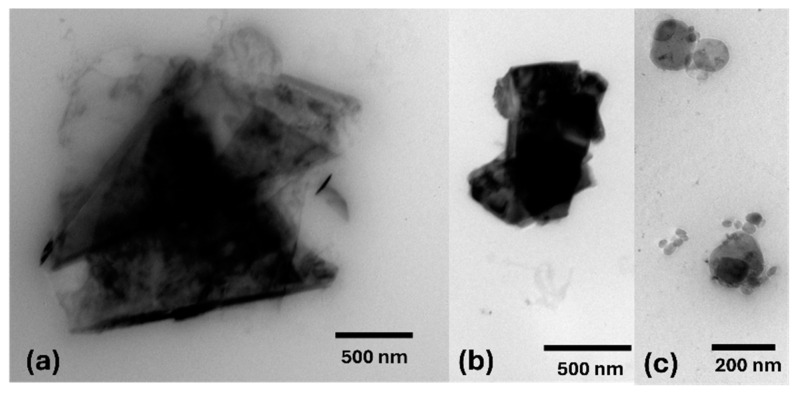
TEM images of (**a**) graphene, (**b**) molybdenum disulfide and (**c**) hexagonal boron nitride flakes exfoliated in cyrene.

The particle size of the particles using the DLS technique agrees with the particle size measured by the TEM technique, as shown in Figure 2. The pictures of the particles exfoliated in polymeric solution were not shown here since the presence of polymer partially covered the particles, so it became difficult to visualize them by the TEM technique.

The zeta potential (ζ) of particles dispersed only in cyrene was also determined, and the results are shown in Table 3. The $\left|\zeta \right|$ exceeding 30 mV for the hBN particles indicates moderate electrostatic stability of the suspension. In contrast, MoS_2_ and GNP particles, with $\left|\zeta \right|$ over 40 mV, exhibit good electrostatic stability [37].

When polymers are present in the suspension, the zeta potential of the particles is significantly influenced, potentially neutralizing the electric charge on the particle surface ($\left|\zeta \right|\sim \;0$ mV) [38]. For suspensions with dissolved polymer, $\left|\zeta \right|$ values were found to be ≲10 mV, reflecting that the stability of the suspended particles is ensured through the steric mechanism [39].

### 3.2. Shear Rheological Characterization

**Figure 3:**
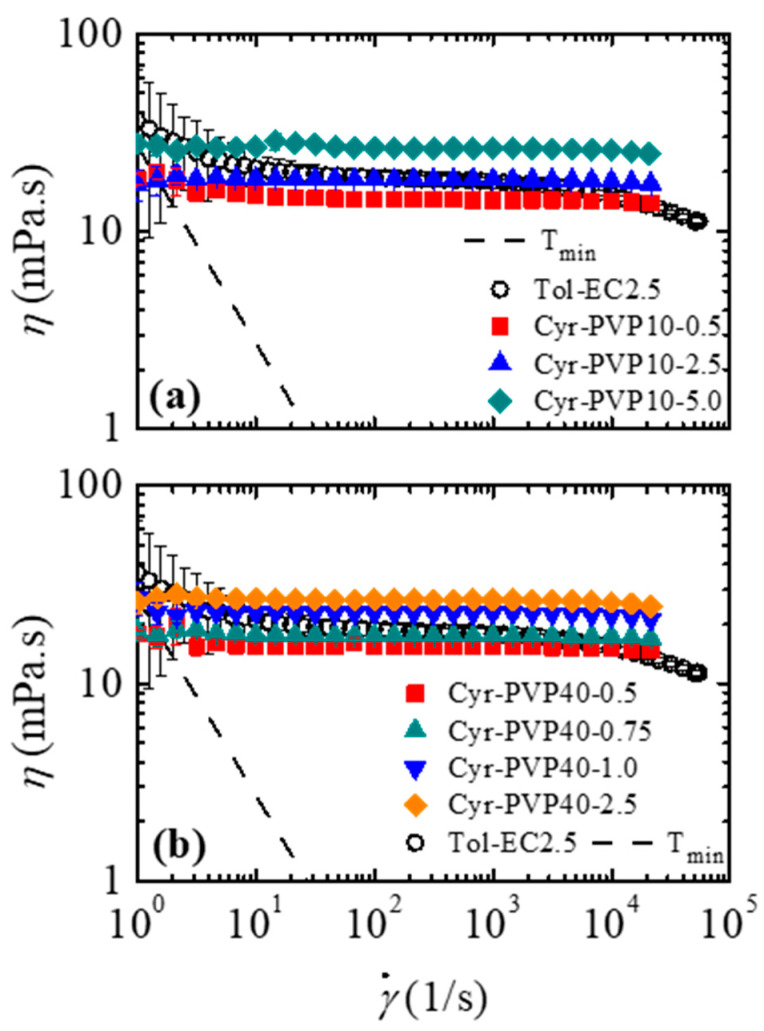
Comparison of shear viscosity curve of Tol-EC2.5 with shear viscosity curves for different PVP concentrations. Molecular weight of PVP: (**a**) 10,000 g/mol and (**b**) 40,000 g/mol. The dashed line represents the minimum torque ($T_{min}$) line. All shear viscosity values above the $T_{min}$ line are credible values, otherwise they are affected by the $T_{min}$ induced by the rotational rheometer.

Before the formulation of 2D inks, it is important to establish the concentration of polyvinylpyrrolidone that promotes a shear viscosity curve similar to the curve obtained for the Tol-EC2.5 solution studied previously [30]. Figure 3 shows how the PVP concentration affects the shear viscosity for different molecular weights: 10,000 g/mol (PVP10) and 40,000 g/mol (PVP40).

Regardless of the molecular weight, it can be seen that the viscosity of the PVP solution is practically constant and independent for a wide range of shear rates ($\dot{\gamma }<10^{4}$ s^−1^). For shear rates above $10^{4}$ s^−1^, the polymer solution shows a slight shear-thinning behavior. This behavior was also observed when PVP of different molecular weights was dissolved in distilled water, ethanol and heptanol [40,41,42]. Furthermore, Franco et al. [11] reported similar behavior when a PVP with a molecular weight of $10^{6}$ g/mol was dissolved in cyrene.

According to Figure 3, the green alternative carrier fluids that can replace the Tol-EC2.5 solution are PVP10 dissolved in cyrene with a concentration of 2.5% *w*/*w* (Cyr-PVP10-2.5) and PVP40 dissolved in cyrene at a concentration of 0.75% *w*/*w* (Cyr-PVP40-0.75). These two solutions have shear viscosity curves similar to the curve obtained for Tol-EC2.5. Although both carrier fluids behave in the same way, the greater the molecular weight of the polymer, the lower the polymer concentration required to provide the same viscosity value. Furthermore, the green carrier fluids have a PVP concentration higher than the overlap concentration (c*), as shown in Table 4. The calculations used to determine the overlap concentration for each molecular weight are available in Section SI-1 in Supplementary Materials.

The presence of 2D nanoparticles may also affect the rheological behavior of cyrene after the exfoliation process. Figure 3 shows that cyrene behaves like a Newtonian fluid, which has a zero-shear viscosity of 13.23 ± 0.27 mPa·s. This value is very close to the viscosity value of 14.5 mPa·s reported in the literature [13,18]. The addition of nanoparticles shows that the rheological behavior of the suspension is affected by the chemical composition of the nanoparticles. When the exfoliated particles are graphene (GNPs), Figure 3 shows a marked shear-thinning at low shear rates ($\dot{\gamma }<10^{3}$ s^−1^), followed by a plateau at high shear rates ($\dot{\gamma }>10^{3}$ s^−1^). This pronounced shear-thinning was also observed by Franco et al. [11] and was justified to be based on a structural reorganization of the particles during flow.

**Figure 4:**
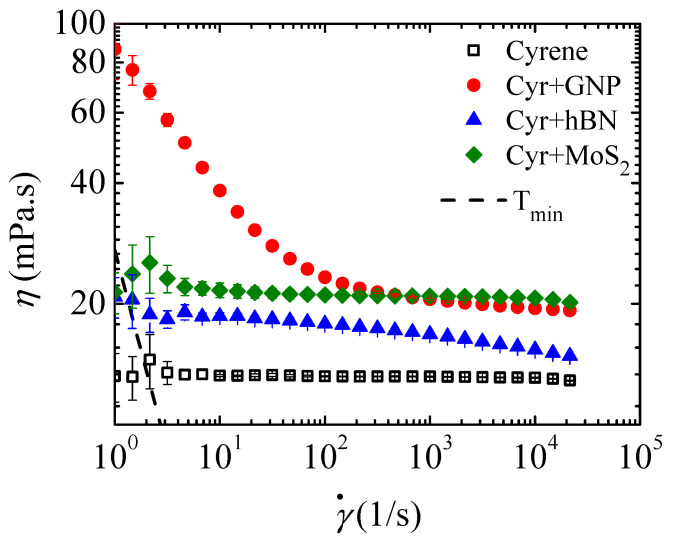
Shear viscosity curves for cyrene (Cyr); and graphene nanoplatelets (GNPs), hexagonal boron nitride (hBN) and molybdenum disulfide (MoS_2_) dispersed in cyrene. The dashed line represents the minimum torque ($T_{min}$) line. All shear viscosity values above the $T_{min}$ line are credible values, otherwise they are affected by the $T_{min}$ induced by the rotational rheometer.

When the exfoliated particles are MoS_2_, the shear viscosity of the Cyr+MoS_2_ suspension remains constant and independent of the applied shear rate, which differs significantly from the rheological behavior of graphene (Cyr+GNP) and hBN (Cyr+hBN) suspensions. In the case of the Cyr+hBN suspension, there is a small plateau in the shear viscosity at low shear rates ($\dot{\gamma }<10^{2}$ s^−1^), followed by smooth shear-thinning (Figure 4).

According to Claypole et al. [43], the viscosity of a particle suspension is strongly dependent on the particle volume fraction, particle size distribution, particle shape, interactions between particles and particle–fluid interactions, and the surface properties of the particles. For nonsymmetric particles, such as rods, disks, sheets, etc., the viscosity of a suspension is strongly affected by the particle shape (aspect ratio) and particle concentration. Rueda et al. [44] and Barnes et al. [45] found that higher aspect ratio particles lead to higher values of shear viscosity and intrinsic viscosity and lower values of maximum packing volume fraction compared to the spherical particles for the same volume fraction. Despite GNP, hBN and MoS_2_ being considered 2D particles with thicknesses of a few nanometers and aspect ratios much higher than 1, the reality is that they have not been perfectly exfoliated in the suspensions, as can be observed from the average particle size on the order of a few hundred nanometers (Table 2). In the determination of average particle size using a Zetasizer, the calculations typically assume that the particles are spherical; thus, the reported size is the hydrodynamic diameter, which is the diameter of a sphere that has the same diffusion coefficient as the particle being measured. Therefore, it is expected that the suspensions with larger hydrodynamic particle sizes will provide higher viscosity values. Figure 4 shows that the particles with the largest hydrodynamic diameter are GNPs, followed by MoS_2_, and finally, hBN particles. This order corresponds to the viscosity curves at the same particle concentration.

**Figure 5:**
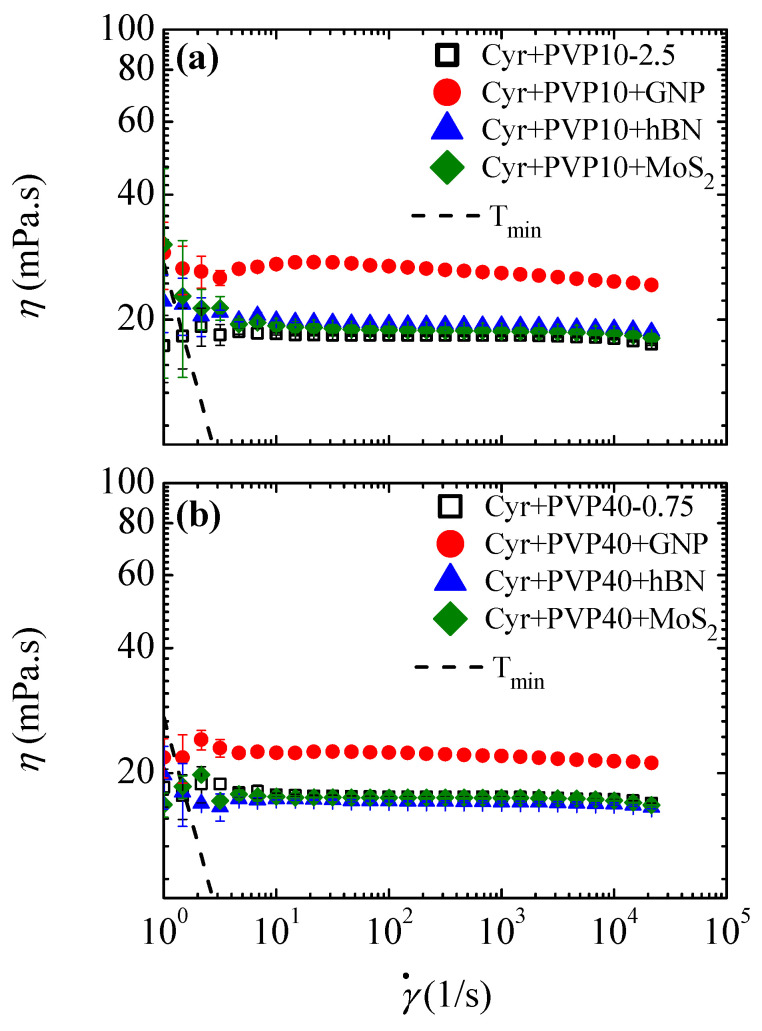
Shear viscosity curves for GNP, hBN and MoS_2_ dispersed in (**a**) 2.5% *w*/*w* of PVP10 dissolved in cyrene (Cyr+PVP10-2.5) and (**b**) 0.75% *w*/*w* of PVP40 dissolved in cyrene (Cyr+PVP40-0.75). The dashed line represents the minimum torque ($T_{min}$) line. All shear viscosity values above the $T_{min}$ line are credible values, otherwise they are affected by the $T_{min}$ induced by the rotational rheometer.

Figure 5 shows that PVP attenuated shear-thinning for low shear rates when the particles were exfoliated in the presence of polymer. This is particularly evident in the case of GNP particles (Cyr+PVP10+GNP and Cyr+PVP40+GNP). The presence of PVP also delayed shear-thinning at high shear rates for hBN inks (Cyr+PVP10+hBN and Cyr+PVP40+hBN). In relation to the MoS_2_ inks (Cyr+PVP10+ MoS_2_ and Cyr+PVP40+ MoS_2_), the shear viscosity remained constant and independent for a wide range of shear rates, reproducing the rheological behavior observed for the Cyr+MoS_2_ suspension. Therefore, the presence of PVP causes the inks to behave like Boger fluids. Boger fluids with a weak elasticity component can be an advantage compared to Newtonian fluids for printing techniques [46,47,48].

### 3.3. Extensional Rheological Characterization

In printing processes, it is important to know how the filament breaks to release a droplet when a fluid is exposed to elongational flow. That information can be extracted from the experiments in the CaBER device, where the time evolution of the minimum radius of the liquid bridge is measured during the shear-free uniaxial extensional flow conditions. The mathematical analysis of the filament thinning process allows us to determine the dominant forces for each ink formulation. In this way, different regimes can be identified, i.e., inertio-capillary (IC), visco-capillary (VC) and inertio-viscous-capillary (IV) regimes (Table 5).

In this work, the inks are composed of functional particles and behave, from a rheological point of view, like a suspension, and the dynamics of pinch-off and filament thinning are very similar to the behavior observed in [49,50,51,52]. Initially, the suspension behaves like bulk fluid, and the thinning process is not affected by the presence of particles. When the filament diameter approaches the size of the larger particles, the fluctuations of particle density start to appear, and the thinning rate accelerates in the region poor in larger particles. In this region, the larger particles are pushed towards the neck of the filament, accelerating the thinning process until it breaks. This acceleration of the thinning process is also caused by a decrease in the viscosity of the fluid promoted by the reduction in the particle concentration in the poor particle region.

**Figure 6:**
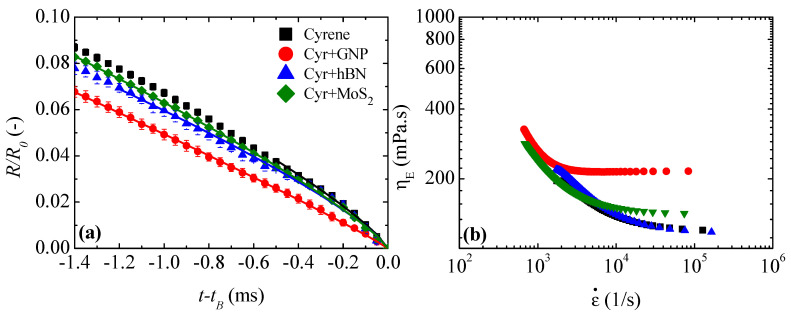
(**a**) Time evolution of the minimum filament radius of cyrene and GNP, hBN and MoS_2_ dispersed in cyrene (Cyr). (**b**) The dependency of the extensional viscosity ($\eta_{E}$) of the extension rate ($\dot{\varepsilon }$) of cyrene and GNP, hBN and MoS_2_ dispersed in cyrene. Solid lines represent the fit of the power law equation (Equation (1)) during the IV regime.

Figure 6a shows the time evolution of the minimum filament radius of cyrene and particles (GNP, hBN and MoS_2_) dispersed in cyrene. The presence of particles delays the thinning rate of the filament when it is stretched, and the suspensions have a longer lifetime than cyrene. Moreover, the presence of particles anticipates the end of the inertio-capillary (IC) regime, where the inertia and capillary forces are dominant and control the dynamics of the filament thinning. In the IC regime, the filament radius decays following a power law as follows:

(1) $$\frac{R_{min}}{R_{0}}\left(t\right)=A{\left(t_{B}-t\right)}^{n}$$

where $R_{min}$ is the minimum radius of the filament, $R_{0}$ represents the initial radius of the filament, A and n are fitting parameters and $t_{B}$ is the time that the filament needs to break up. Inertia and capillary forces are dominant, and the exponent n is 2/3. For the case of cyrene, the IC regime is dominant until the last 0.5 ms of the filament lifetime since the Ohnesorge (Oh) number is lower than 0.2077. For $t-t_{B}<-0.5$ ms, there is a transition from the inertio-capillary regime to the visco-capillary (VC) regime since Oh>0.2077. In the VC regime, the filament radius decreases linearly as

(2) $$R_{min}\left(t\right)=\frac{2X-1}{6}\frac{\sigma }{\eta }(t_{B}-t)$$

where X is a constant, and σ and η are the surface tension and shear viscosity of the fluid, respectively. However, this equation does not fit the experimental curves present in Figure 6, unlike Equation (1). For pure cyrene, the exponent n takes the value of 0.8066, which is higher than 2/3 and lower than 1. When this happens, the inertia, viscous and capillary forces are dominant and control the dynamics of the capillary breakup [53] following the power law described in Equation (1). Some works proposed that the VC regime occurs for Oh>1, whereas for 0.2077<Oh<1, it corresponds to the transition stage where the inertia force decreases to negligibility, coupled with an increase in the viscous force [51,52,53,54,55]. Considering the Oh interval, Table 6 shows that the filament thinning process is controlled by the IC regime when Oh<0.2077 and/or by the inertio-visco-capillary (IV) regime when 0.2077<Oh<1 for cyrene and particles dispersed in cyrene. Table 6 also shows the values of parameters A and n that fit the experimental curves in Figure 6a with Equation (1).

The extensional viscosity ($\eta_{E}=\sigma /(\dot{\varepsilon }R_{min})$) curves in Figure 6b show that the extensional viscosity decreases with the extension rate ($\dot{\varepsilon }(t)=-\frac{2}{R_{min}\left(t\right)}\cdot \frac{dR_{min}}{dt}(t)$) until it reaches a constant viscosity plateau at high extension rates ($\dot{\varepsilon }>10^{4}$ s^−1^). Knowing that $\;\dot{\gamma }=\dot{\varepsilon }/\sqrt{3}$, there is a plateau of shear viscosity and extensional viscosity for the equivalent range of shear and extension rates for all fluids except Cyr+hBN, where a gradual reduction in shear viscosity is observed due to the shear-thinning behavior of this suspension (Figure 4 and Figure 6). For cyrene, GNP and MoS_2_ suspensions, the presence of these two plateaus shows that the fluids exhibit a Newtonian-like behavior. However, the Trouton ratio ($Tr=\eta_{E}/\eta_{0}$) for cyrene and GNP and MoS_2_ suspensions is between 2 and 3.5 times higher than the Trouton ratio defined for Newtonian fluids subjected to uniaxial extensional flow (Tr=3). However, a Trouton ratio of 3 would be expected for pure cyrene, as it is a Newtonian fluid. It was not possible to obtain this ratio since it was not possible to eliminate the inertia effects felt by the fluid in the experimental setup used in this work.

**Figure 7:**
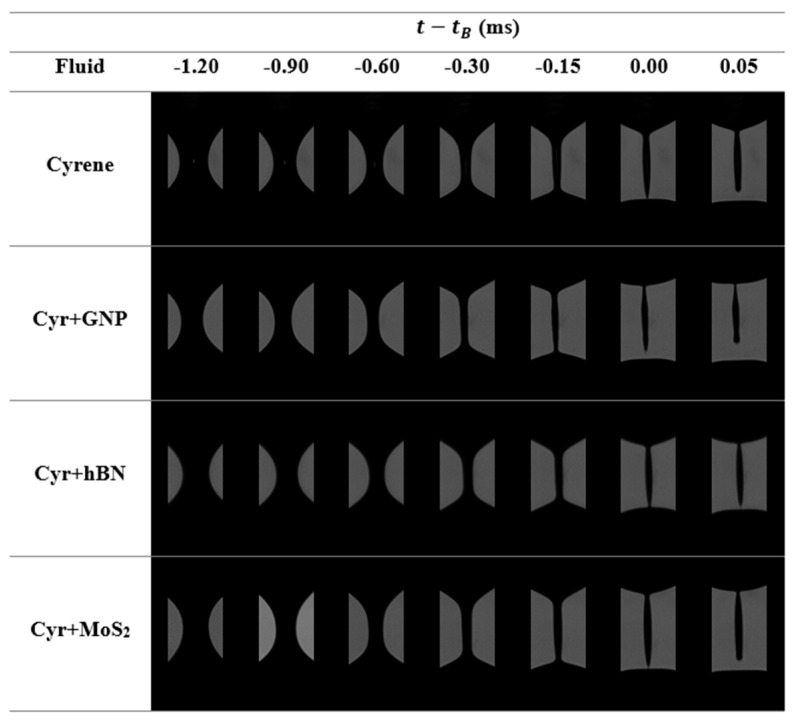
Photographic record of the thinning process for the suspensions that have cyrene as a carrier fluid.

The last snapshots of the filament thinning process for the cyrene and 2D particles dispersed in cyrene are represented in Figure 7. The pinch-off dynamics (Figure 7) reinforces the presence of inertia during the filament breakup since it was not possible to obtain a perfect pinch-off at the moment of the filament breakup, i.e., simultaneous breakage of the small filaments connecting the ends of the drops and the pinch.

**Figure 8:**
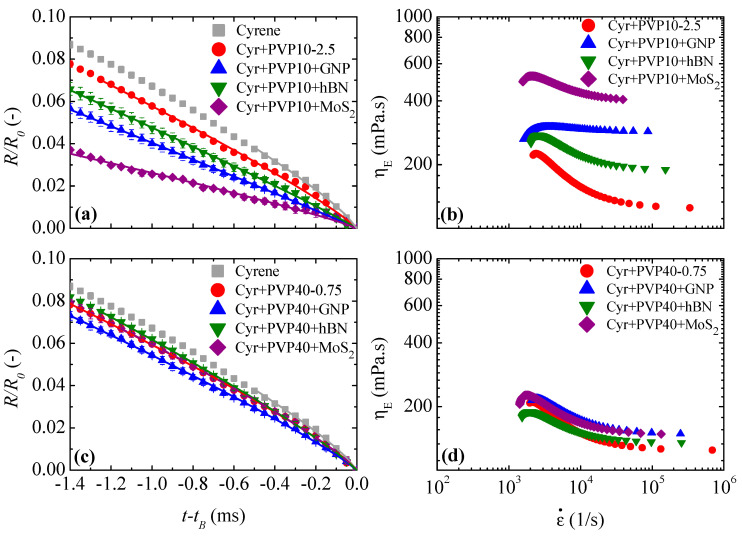
Time evolution of the minimum filament radius and the dependency of the extensional viscosity ($\eta_{E}$) of extension rate ($\dot{\varepsilon }$) for GNP, hBN and MoS_2_ dispersed in (**a**,**c**) 2.5% *w*/*w* of PVP10 dissolved in cyrene and (**b**,**d**) 0.75% *w*/*w* of PVP40 dissolved in cyrene. Solid lines represent the fit of the power law equation (Equation (1)) during the IV regime.

When the carrier fluid is Cyr+PVP10 with a concentration of 2.5% *w*/*w*, the filament lifetime is longer compared to the lifetime of the filament composed only of cyrene (Figure 8a). However, the addition of nanoparticles speeds up the thinning process, similar to what was observed when these nanomaterials were dispersed in Tol-EC2.5 for the same particle concentration [8]. When the diameter of the filament is close to the size of the largest particles, it creates a buffering effect, promoting the creation of particle-rich and particle-poor regions [49]. The presence of this type of region destroys the homogeneity condition of the suspension. The particle-free regions control the thinning process, which means that $R/R_{0}$ curves of the inks with particles overlap the $R/R_{0}$ curve obtained for the carrier fluid (Cyr+PVP10-2.5) before the filament breaks; see Figure 8a.

For all fluids in Figure 8a, the Ohnesorge number is greater than 0.2077 and less than or close to 1 for the time scale used, which shows that inertial, viscous and capillary forces are dominant and control the dynamics of the filament thinning when it is stretched. Therefore, the IV regime is present in the last 1.4 ms of the filament lifetime, and Equation (1) fits well with the experimental curves shown in Figure 8a. Table 7 shows the fit values for parameters A and n, and the root mean square error was below 0.01 for all experimental data. Regarding the extensional viscosity curves shown in Figure 8b, the extensional viscosity decreases with the extension rate until it stabilizes at high extension rates. The plateau observed in the extensional and shear viscosity curves occurs for the same range of shear and extension rates since $\dot{\gamma }=\dot{\varepsilon }/\sqrt{3}$. These two plateaus indicate a Newtonian behavior of the fluids at high shear and extension rates despite the Trouton ratio being 2–5 times higher than the characteristic Trouton ratio for Newtonian fluids (Tr=3).

**Figure 9:**
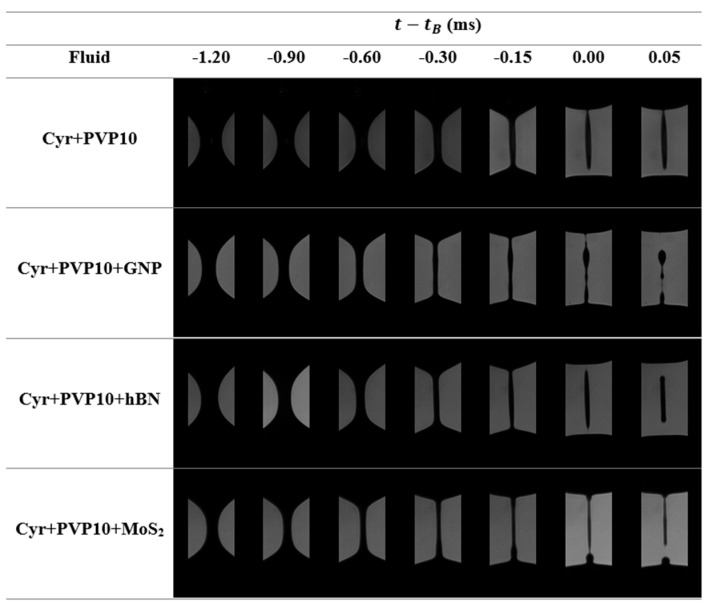
Photographic record of the thinning process for the suspensions that have 2.5% *w*/*w* of PVP10 dissolved in cyrene as carrier fluid.

When the filament thins, the presence of particle-rich and particle-poor regions can lead to the creation of beads-on-a-string (BOAS) structures composed of one elongated droplet or several spherical droplets, as can be seen in Figure 9.

**Figure 10:**
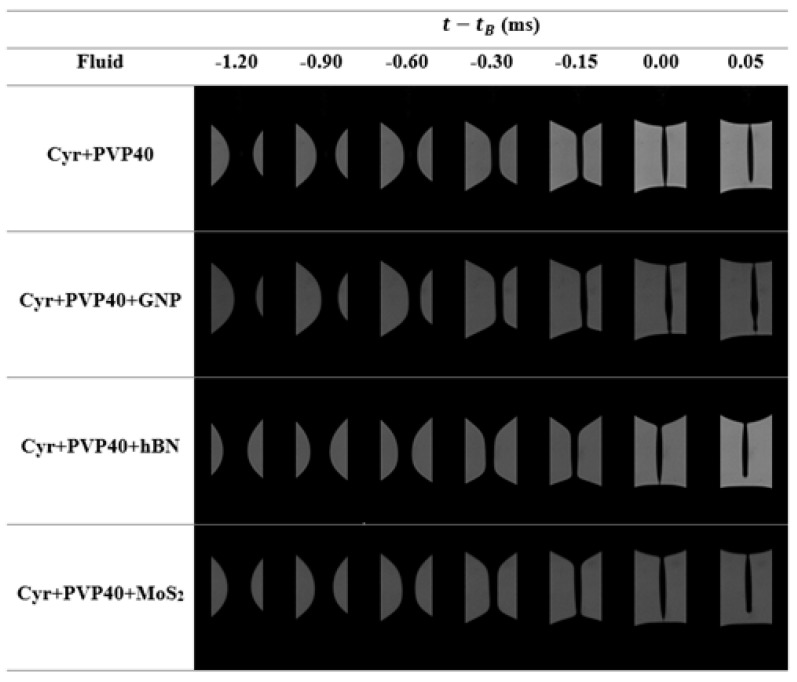
Photographic record of the thinning process for the suspensions that have 0.75% *w*/*w* of PVP40 dissolved in cyrene as carrier fluid.

When the carrier fluid is Cyr+PVP40 at a concentration of 0.75% *w*/*w*, Figure 8c shows a slight slowdown in the thinning process when the polymer is present. Moreover, the addition of particles does not significantly affect the thinning process and filament lifetime. However, it is possible to observe an overlap of the $R/R_{0}$ curves of the inks with and without particles for an extended time range. As happened for Cyr+PVP10 inks, the inertio-viscous-capillary regime controls the thinning process, Equation (1) fits well with the experimental curves shown in Figure 8c and the fitted parameters can be viewed in Table 7. Regarding the extensional viscosity, the inks with PVP40 show a plateau at high extension rates, which is accompanied by a plateau of the shear viscosity for an equivalent shear rate range, which indicates the Newtonian-like behavior of these fluids at high extension and shear rates. Figure 10 shows that the particle-poor regions are located at the ends of the droplets, while the elongated droplet region is particle-rich.

Knowing that the concentrations of PVP10 and PVP40 used in this work are higher than the overlap concentration, one would expect to see an exponential decay of the $R/R_{0}$ during the thinning process due to the elastic component of the fluid. However, this situation could not be observed in the polymer inks. According to Gaillard et al. [56], the flow history of the fluid can affect the formation of an elasto-capillary regime of a viscoelastic filament and, subsequently, the determination of the apparent extensional relaxation time. In that work, they suggested a dependence of the relaxation times with the initial gap separation ($h_{0}$); they stated that low $h_{0}$ values may result in less elastic behaviors, but further research is required to be conclusive. According to Clasen et al. [57], inertial and gravitational effects are only minimized in CaBER when a cylindrical liquid bridge pinned to two endplates ($h_{0}$) is below the capillary length ($l_{cap}$), as follows:

(3) $$h_{0}\leq l_{cap}=\sqrt{\frac{\sigma }{\rho g}}$$

where g is the gravitational acceleration. Considering the properties of cyrene, the inertial and gravitational effects would be minimized if $h_{0}\leq 1.85$ mm. In addition, in CaBER it is necessary to ensure that the initial aspect ratio ($\Lambda_{0}=h_{0}/D_{plate}$) is greater than or equal to 0.5 [57,58], which would imply using a plate diameter less than 4 mm, which is not commercially available.

Therefore, the relaxation time could not be determined. However, considering the image acquisition rate (20,000 fps), it can be concluded that the apparent relaxation time is shorter than 50 µs.

### 3.4. Estimated Analysis of 2D Inks for Inkjet Printing and EHD Jet Printing

After the rheological characterization of the new green 2D inks as alternatives to the 2D inks based on toluene studied previously [8,30,31,32], it is necessary to evaluate whether these inks can be used in inkjet printing or EHD jet printing techniques.

In inkjet printers, more specifically drop-on-demand (DoD) inkjet printers, the printing stability region is based on the fluid’s properties [19,24,59]. This stability region consists of a dimensional analysis based on the Ohnesorge number [19], as follows:

(4) $$Oh=\frac{\sqrt{We}}{Re}=\frac{\eta }{\sqrt{\sigma \rho D_{nozzle}}}$$

where $We=\rho D_{nozzle}v_{fluid}^{2}/\sigma$ is the Weber number, $Re=\rho D_{nozzle}v_{fluid}/\eta$ is the Reynolds number, $v_{fluid}$ is the velocity of the injected fluid and $D_{nozzle}$ is the nozzle diameter. An ink is in the printing stability region when 0.1≤Oh≤1 and 2≤We≤25 [19]. If Oh<0.1, the inks do not have enough energy to create droplets, and when Oh>1, the capillary force hinders the droplet ejection since the ink is too viscous. For We<2, the ink promotes the formation of satellite droplets, which is undesirable for inkjet printing, and the disintegration of the ink jet in several droplets (atomization) occurs when We>25 due to the exaggerated velocity at which the ink is ejected [19].

The minimum and maximum nozzle diameters, as well as the respective minimum and maximum flow rates in the printing stability region for each ink, have been determined theoretically based on the Oh number limits; the minimum and maximum nozzle diameters are determined as follows:

(5) $$Oh=1\to {D_{nozzle}}_{min}=\frac{\eta^{2}}{\rho \sigma }$$

(6) $$Oh=0.1\to {D_{nozzle}}_{max}=100\frac{\eta^{2}}{\rho \sigma }$$

Knowing that the ejected flow rate is $Q=\pi D_{nozzle}^{2}v_{fluid}/4$, then the We number can be calculated as a function of the flow rate as

(7) $$We=\frac{16\rho Q^{2}}{\pi^{2}\sigma D_{nozzle}^{3}}$$

So, the minimum and maximum flow rates occur when

(8) $$We=2\to Q_{min}=\frac{\pi }{4}{\left(\frac{2\sigma }{\rho }D_{nozzle}^{3}\right)}^{1/2}$$

(9) $$We=25\to Q_{max}=\frac{5\pi }{4}{\left(\frac{\sigma }{\rho }D_{nozzle}^{3}\right)}^{1/2}$$

As most inks behave similarly to weakly elastic Boger fluids, where the shear viscosity remains constant and independent of the shear rate applied for a wide $\dot{\gamma }$ range, zero-shear viscosity ($\eta_{0}$) was used in Equations (5) and (6). The values of density, surface tension and zero-shear viscosity used for each ink can be found in Table 8. However, Cyr+GNP ink showed marked shear-thinning at low shear rates ($\dot{\gamma }≲100$ s^−1^), which meant that zero-shear viscosity cannot be used in Equations (5) and (6). As the minimum shear rate applied in DoD inkjet printing corresponds to the condition Oh=0.1 and We=2, it was found that $\dot{\gamma }=362$ s^−1^. For this shear rate, the shear viscosity is 21.27 mPa·s.

**Figure 11:**
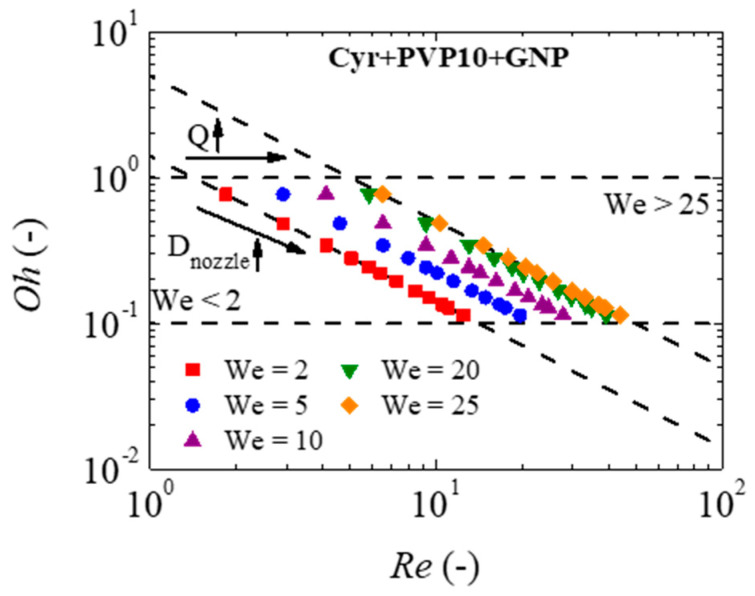
Influence of nozzle diameter and flow in the printing stability region of Cyr+PVP10+GNP ink.

Table 9 shows the minimum and maximum nozzle diameter and flow rate values for each ink in order to respect the limits imposed in the printing stability region. Figure 11 shows how the nozzle diameter and flow rate used affect the Oh and We numbers for Cyr+PVP10+GNP ink.

The data from Table 8 and Table 9 indicate that viscosity is the fluid property with the greatest impact on the maximum nozzle diameter in DoD inkjet printing applications. However, the resulting droplet diameter in this type of printing technique, regardless of the flow rate applied, depends solely on the fluid’s properties (density and surface tension) and the nozzle diameter. Although it was not possible to measure the relaxation time under extensional flow, we will assume that 50 µs or lower introduces a negligible effect on the droplet breakup dynamics and its size distribution. In drop-on-demand inkjet printing, there is no universal equation that allows us to predict the diameter of the drop depending on the diameter of the nozzle and the properties of the fluid. So far, there are several empirical equations for predicting the droplet diameter, but they depend on the experimental conditions and the physical mechanism used to produce the main droplet, such as gravity, piezoelectricity and heat. As a result, several published works provide evidence that the droplet diameter follows the following trend [30,60,61,62,63]:

(10) $$D_{drop}\;\propto \alpha D_{nozzle}$$

where α is an experimental constant and depends on the fluid properties and the operating conditions used during the DoD inkjet printing. Several works show that this constant varies between 1 and 10 [30,60,61,62,63]. Based on this range of α values, a droplet diameter estimate region was defined considering the minimum and maximum α values in order to be able to compare with the droplet diameters produced by electrohydrodynamic (EHD) jet printing.

Regardless of the nozzle diameter or the flow rate applied, the droplet diameter is always greater than the nozzle diameter used in the DoD application. According to Franco et al. [64], the maximum particle size should be 1/10th of the nozzle diameter used in inkjet printing and EHD jet printing. Considering the largest particles are graphene dispersed in cyrene with an average diameter of 1863 ± 40 nm (Table 2), the minimum nozzle diameter to be used in both printing techniques should be 10 times the size of the largest particles, which is 18.63 μm, to avoid clogging problems in the nozzles. To simplify the calculations and ensure that the jet diameter at the tip of the Taylor cone is not influenced by the nozzle diameter, we have decided to used a nozzle diameter of 20 μm.

EHD jet printing has proved to be an alternative to DoD inkjet printing, allowing the production of droplets smaller than the nozzle diameter used in the system. This is only possible due to the application of an electric field, which causes the electric charges present in the fluid to move towards the air/liquid interface, forcing the meniscus to deform according to a structure similar to a Taylor cone [28]. Gañán-Calvo et al. proposed a set of scaling laws that allow us to predict the diameter of the droplet ejected at the tip of the Taylor cone [65]. But to do this, it is first necessary to determine the characteristic diameter ($d_{0}$) and the characteristic flow rate ($Q_{0}$) based on the properties of the fluid [65], as follows:

(11) $$d_{0}={\left(\frac{\sigma \varepsilon_{0}^{2}}{\rho \kappa^{2}}\right)}^{1/3},\;Q_{0}=\frac{\sigma \varepsilon_{0}}{\rho \kappa }$$

where $\varepsilon_{0}$ is the vacuum permittivity and κ is the electrical conductivity of the fluid. Table 8 shows the electrical conductivity values determined experimentally for each ink. In Supplementary Materials, Table S2 shows the characteristic values of $d_{0}$ and $Q_{0}$ for each ink. In terms of electrical conductivity, it can be observed that the addition of polymer and particles improves the electrical conductivity of cyrene.

According to Gañán-Calvo et al. [66], the diameter of the droplet ejected at the tip of the Taylor cone is scaled as

(12) $$D_{drop}\sim {\left(\frac{\rho \varepsilon_{0}}{\sigma \kappa }Q^{3}\right)}^{\frac{1}{6}}\approx {\left(\frac{\sigma }{\rho }\right)}^{\frac{1}{3}}{\left(\frac{\varepsilon_{0}}{\kappa }\right)}^{\frac{2}{3}}{\left(\frac{Q}{Q_{0}}\right)}^{\frac{1}{2}}$$

**Figure 12:**
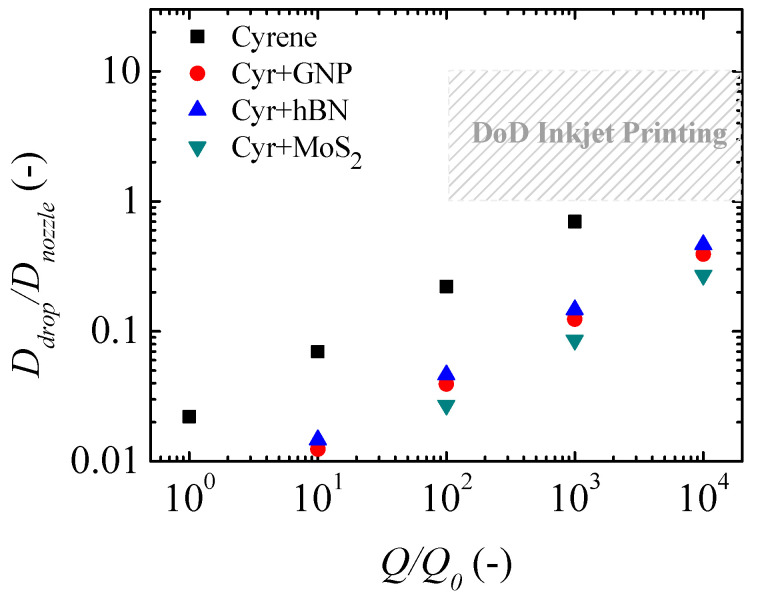
Normalized droplet diameter ($D_{drop}/D_{noz}$) as a function of normalized flow rate ($Q/Q_{0}$) for inks without polymer. The open symbols represent the estimations for DoD inkjet printing, in contrast to filled symbols representing the estimations for EHD jet printing.

**Figure 13:**
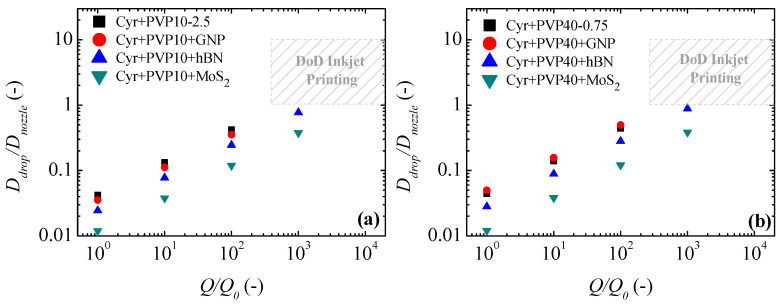
Normalized droplet diameter ($D_{drop}/D_{noz}$) as a function of normalized flow rate ($Q/Q_{0}$) for inks with (**a**) PVP10 and (**b**) PVP40. The open symbols represent the estimations for DoD inkjet printing, and the filled symbols represent the estimations for EHD jet printing.

Figure 12 and Figure 13 show the estimated ratio between the droplet diameter and the nozzle diameter ($D_{drop}/D_{nozzle}$) as a function of the $Q/Q_{0}$ ratio. In this comparative analysis between the droplet diameter predicted for DoD inkjet printing and EHD jet printing, a nozzle diameter of 20 μm was used. This diameter ensures that the ratio between the characteristic diameter (*d*_0_) and the nozzle diameter (*D_nozzle_*) is less than 10^-2^, avoiding the dependence of the nozzle diameter on the jet diameter at the tip of the Taylor cone [67].

Regardless of the concentration and molecular weight of the polymer used, the $D_{drop}/D_{nozzle}$ ratio in DoD inkjet printing is always higher than the $D_{drop}/D_{nozzle}$ ratio obtained for EHD jet printing. Using the range of flow rates allowed in the DoD printers for the 100 μm nozzle to respect the limits of the printing stability region, Figure 12 and Figure 13 show that the $D_{drop}/D_{nozzle}$ ratio in DoD applications can be up to two orders of magnitude higher compared to the $D_{drop}/D_{nozzle}$ ratio obtained for EHD printing.

## 4. Conclusions

This study successfully demonstrates that eco-friendly 2D inks can be formulated based on cyrene, presenting a sustainable alternative to traditional toluene-based formulations for high-precision printing techniques, such as inkjet and electrohydrodynamic (EHD) jet printing. Cyrene demonstrated excellent exfoliation capabilities for graphene nanoplatelets (GNPs), hexagonal boron nitride (hBN) and molybdenum disulfide (MoS_2_), achieving reduced particle sizes and improved dispersion stability. These characteristics not only enhance the performance of 2D inks but also reduce the risk of nozzle clogging, a critical challenge in advanced printing applications.

The incorporation of polyvinylpyrrolidone (PVP) into the cyrene-based system significantly influenced the rheological, electrical and extensional properties of the inks, showcasing their potential for various high-precision printing techniques. The incorporation of polyvinylpyrrolidone (PVP) into the cyrene-based formulations effectively mitigated shear-thinning behavior and improved viscosity control, resulting in Boger fluid-like properties that are advantageous for achieving uniform droplet formation. Furthermore, this study provides valuable insights into the extensional rheology of these inks, identifying distinct filament thinning regimes, including inertio-capillary (IC), visco-capillary (VC), and inertio-viscous-capillary (IV), which are critical for optimizing printability and droplet stability.

Importantly, the inks exhibit Newtonian-like behavior at high shear and extension rates, ensuring compatibility with the operational conditions of both DoD inkjet and EHD jet printing. The comparative analysis also highlights that while DoD printing results in larger droplet sizes, EHD jet printing enables droplet formation at sub-nozzle diameters, offering complementary advantages for different applications.

From a sustainability perspective, cyrene’s non-toxic, renewable origin and high performance position it as a compelling replacement for toluene and other petroleum-based solvents. This work establishes a robust framework for the development of greener 2D ink systems tailored to emerging applications in printed electronics, flexible devices, and energy storage technologies.

Future research should focus on scaling these formulations for industrial production, exploring their behavior across diverse substrates, and further refining rheological properties to optimize performance in ultra-high-resolution printing.

## Figures and Tables

**Table 1:** Surface tension (σ) of the 2D particles and liquids used in liquid-phase exfoliation [8,14].

	Graphene	hBN	MoS_2_	NMP	DMF
σ (mN/m)	44.8	46.5	35–37	41	35.2

**Table 2:** Average particle size determined from the DLS technique for each particle dispersed in three different dispersant mediums.

	Average Particle Size (nm)		
Carrier Fluid	GNP	hBN	MoS_2_
Cyrene	1863 ± 40	171.5 ± 4.0	638.5 ± 49.7
Cyr-PVP10-2.5	1389 ± 476.5	193.6 ± 2.0	732.5 ± 65.7
Cyr-PVP40-0.75	1386 ± 103	147 ± 1.9	243.2 ± 8.9

**Table 3:** Zeta potential (ζ) of the particles when dispersed in three different dispersant media.

	ζ (mV)		
Carrier Fluid	GNP	hBN	MoS_2_
Cyrene	41.3 ± 5.4	−36.1 ± 1.3	−44.8 ± 1.3
Cyr-PVP10-2.5	−5.3 ± 4.9	3.4 ± 3.9	1.7 ± 2.2
Cyr-PVP40-0.75	17.1 ± 4.6	3.2 ± 0.6	6.6 ± 1.5

**Table 4:** Molecular weight (MW), concentration ($c_{PVP}$) and overlap concentration ($c^{*}$) of PVP for the green carrier fluids used in this work.

	Cyr-PVP10-2.5	Cyr-PVP40-0.75
MW (g/mol)	$10\times 10^{3}$	$40\times 10^{3}$
$c_{PVP}$ (% *w*/*w*)	2.5	0.75
$c_{PVP}$ (g/cm^3^)	$31.10\times 10^{-3}$	$9.37\times 10^{-3}$
$c^{*}$ (g/cm^3^)	$12.00\times 10^{-3}$	$4.02\times 10^{-3}$

**Table 5:** Summary of the different regimes that can be identified by means of the filament thinning process undergone in the CaBER device and their implications for printing processes.

**Regime**	Thinning Behavior	Dominant Forces	Implication for Printing
IC	$R_{min}\left(t\right)\propto {\left(t_{B}-t\right)}^{2/3}$	Inertial and Capillary	Suitable for high-speed printing Risk of satellite droplet formation
IV	$R_{min}\left(t\right)\propto {\left(t_{B}-t\right)}^{n}$ being n∈[2/3,1]	Inertial, Viscous and Capillary	Stable printing Balances speed and precision
VC	$R_{min}\left(t\right)\propto \left(t_{B}-t\right)$	Viscous and Capillary	Best suited for high precision and uniform droplets Fluid may fail to form droplets

**Table 6:** The Ohnesorge (Oh) number and the fitted parameters A and n of Equation (1) for cyrene and particles dispersed in cyrene.

Fluid	Oh (-)	A (s^−n^)	n (-)
Cyrene	0.137–0.524	0.0351	0.8066
Cyr+GNP	0.243–1.147	0.0621	0.9328
Cyr+hBN	0.197–0.741	0.0289	0.7948
Cyr+MoS_2_	0.226–0.890	0.0371	0.8228

**Table 7:** The Ohnesorge (Oh) number and the fitted parameters A and n of Equation (1) for the inks with polymer.

Fluid	Oh (-)	A (s^−n^)	n (-)
Cyr+PVP10-2.5	0.200–0.856	0.0475	0.8715
Cyr+PVP10+GNP	0.380–1.927	0.0680	0.9750
Cyr+PVP10+hBN	0.261–1.249	0.0630	0.9402
Cyr+PVP10+MoS_2_	0.436–1.893	0.0231	0.8810
Cyr+PVP40-0.75	0.210–0.849	0.0389	0.8386
Cyr+PVP40+GNP	0.277–1.200	0.0475	0.8801
Cyr+PVP40+hBN	0.198–0.844	0.0503	0.8701
Cyr+PVP40+MoS_2_	0.196–0.795	0.0393	0.8396

**Table 8:** Density (ρ), surface tension (σ), shear viscosity (η) and electrical conductivity (κ) for inks studied in this work.

Fluid	ρ (kg/m^3^)	σ (mN/m)	η (mPa·s)	κ (·10^5^ S/m)
Cyrene	1250	42.83 ± 0.01	13.20 ± 0.30	1.57 ± 0.01
Cyr+GNP	1272	41.99 ± 0.05	20.60 ± 1.20	20.60 ± 0.20
Cyr+hBN	1259	40.38 ± 0.03	17.50 ± 1.50	15.83 ± 0.01
Cyr+MoS_2_	1259	41.30 ± 1.30	21.40 ± 1.10	35.97 ± 0.05
Cyr+PVP10-2.5	1267	42.94 ± 0.03	18.30 ± 0.40	6.70 ± 0.30
Cyr+PVP10+GNP	1261	46.44 ± 0.02	26.30 ± 1.10	9.06 ± 0.01
Cyr+PVP10+hBN	1244	46.92 ± 0.01	19.80 ± 0.80	15.92 ± 0.03
Cyr+PVP10+MoS_2_	1269	46.80 ± 0.05	19.00 ± 1.00	46.16 ± 0.09
Cyr+PVP40-0.75	1272	41.72 ± 0.04	17.70 ± 0.40	6.10 ± 0.01
Cyr+PVP40+GNP	1272	41.72 ± 1.01	21.20 ± 0.60	5.11 ± 0.01
Cyr+PVP40+hBN	1272	41.31 ± 0.02	17.20 ± 0.60	12.05 ± 0.02
Cyr+PVP40+MoS_2_	1266	45.10 ± 1.50	17.50 ± 0.60	44.25 ± 0.07

**Table 9:** Minimum and maximum nozzle diameters and flow rates to be applied in the printing stability region for the DoD inkjet printer.

Fluid	${D_{nozzle}}_{min}$ (μm)	$Q_{min}$ (μL/s)	$Q_{max}$ (μL/s)	${D_{nozzle}}_{max}$ (μm)	$Q_{min}$ (μL/s)	$Q_{max}$ (μL/s)
Cyrene	3.27	0.04	0.14	327	38.46	135.96
Cyr+GNP	7.95	0.14	0.51	795	143.17	506.19
Cyr+hBN	6.04	0.09	0.33	604	93.46	330.45
Cyr+MoS_2_	8.81	0.17	0.59	881	166.31	587.98
Cyr+PVP10-2.5	6.14	0.10	0.35	614	98.37	347.79
Cyr+PVP10+GNP	11.78	0.27	0.96	1178	272.62	963.86
Cyr+PVP10+hBN	6.69	0.12	0.42	669	117.94	416.99
Cyr+PVP10+MoS_2_	6.20	0.10	0.37	620	104.16	368.27
Cyr+PVP40-0.75	5.87	0.09	0.32	587	90.52	320.05
Cyr+PVP40+GNP	9.24	0.18	0.63	924	178.68	631.72
Cyr+PVP40+hBN	5.61	0.08	0.30	561	84.18	297.62
Cyr+PVP40+MoS_2_	5.37	0.08	0.29	537	82.44	291.46

## Data Availability

All data generated or analyzed during this study are included in this published article and its Supplementary Information Files.

## Supplementary Materials

The following supporting information can be downloaded at: https://www.mdpi.com/article/10.3390/mi16020130/s1. Table S1. Molecular weight (MW)t, hydrodynamic radius ($R_{H}$), gyration radius ($R_{G}$) and overlap concentration ($c^{*}$) for polyvinylpyrrolidone with different molecular weights. Table S2. Characteristics axial length ($d_{0}$)and flow rate ($Q_{0}$) for EHD experiments. References [68,69,70] are cited in the Supplementary Materials.
