# Supplementary material for: Eco-Friendly Alternatives to Toluene-Based 2D Inks for Inkjet and Electrohydrodynamic Jet Printing Processes: A Rheological Study
Source: Micromachines (Basel). 2025 Jan 23;16(2):130. doi: 10.3390/mi16020130 (PMC11857509; doi:10.3390/mi16020130)
Supplement: Supplementary file 1 [file micromachines-16-00130-s001.zip › micromachines-3381102-supplementary.pdf]

Supplementary Materials

SI-1. Determination of overlap concentration of Polyvinylpyrrolidone

Overlap concentration (c^*) is an important parameter that allows classifying the polymer solution as dilute, semi-dilute and concentrated depending on the ratio between the polymer concentration (c) and the overlap concentration. The overlap concentration for a given polymer is determined as [1]:

$$c^* = \frac{M_w}{\frac{4}{3}\pi R_G^3 N_A} \quad \text{(SI.1)}$$

where M_w and R_G are the molecular weight and gyration radius of a polymer, and N_A is the Avogadro's number ($N_A = 6.02214 \cdot 10^{23}$ mol^{-1}).The gyration radius of the polymer is $8/3\sqrt{\pi}$ of the hydrodynamic radius (R_H) of the polymer [2]. According to Aschi *et al.* [3], the hydrodynamic radius for polyvinylpyrrolidone with molecular weights of 10000 g/mol and 40000 g/mol are 4.6 nm and 10.5 nm, respectively. So, the gyration radius and overlap concentrations for both polymers are shown in **Table S1**.

Table S1. Molecular weight (MW)t, hydrodynamic radius (R_H), gyration radius (R_G) and overlap concentration ($\boldsymbol{c}^*$) for polyvinylpyrrolidone with different molecular weights.

Polymer	M_w (g/mol)	R_H (nm)	R_G (nm)	c^*(g/cm^3)
**PVP10**	10×10^3	4.6	6.92	12.0×10^{-3}
**PVP40**	40×10^3	10.5	15.80	4.02×10^{-3}

SI-2. Characteristic axial length and flow rate for EHD experiments

Table S2. Characteristics axial length (d_0)and flow rate (Q_0) for EHD experiments.

Fluid	d_0 (μm)	Q_0 (μl/h)
**Cyrene**	2.21	69.39
**Cyr+GNP**	0.39	5.11
**Cyr+hBN**	0.46	6.46
**Cyr+MoS_2**	0.27	2.90
**Cyr+PVP10-2.5**	0.84	16.12
**Cyr+PVP10+GNP**	0.71	12.95
**Cyr+ PVP10+hBN**	0.49	7.55
**Cyr+ PVP10+MoS_2**	0.24	2.55
**Cyr+PVP40-0.75**	0.88	17.14
**Cyr+ PVP40+GNP**	0.99	20.45
**Cyr+ PVP40+hBN**	0.56	8.60
**Cyr+ PVP40+MoS_2**	0.24	2.56

References

1. Rubio, M.; Ponce-Torres, A.; Vega, E.J.; Montanero, J.M. Experimental Analysis of the Extensional Flow of Very Weakly Viscoelastic Polymer Solutions. Materials **2020**, 13, doi:10.3390/ma13010192.

2. Tothova, J.; Lisy, V. Intrinsic viscosity of PVP polymers in extremely diluted solutions. **2013**, 13, doi:10.1515/epoly-2013-0122.

3. Aschi, A.; Jebari, M.M.; Gharbi, A. Investigation of Poly(vinyl pyrrolidone) in methanol by dynamic light scattering and viscosity techniques. **2007**, 7, doi10.1515/epoly.2007.7.1.225.
